# Oral Health Knowledge and Experience of Pediatricians and Pediatric Residents in Kuwait: A Nationwide Cross-Sectional Survey Study

**DOI:** 10.1155/2022/2339540

**Published:** 2022-06-06

**Authors:** Abrar Alanzi, Saleh Hajiah, Anfal Faridoun, Aisha Alterkait

**Affiliations:** ^1^Department of Developmental and Preventive Sciences, Faculty of Dentistry, Kuwait University, Kuwait City, Kuwait; ^2^Specialty Dental Centre, Ministry of Health, Kuwait City, Kuwait; ^3^Pediatric Department, Ministry of Health, Kuwait City, Kuwait

## Abstract

**Objective:**

To evaluate the knowledge level and practice of pediatricians and pediatric residents regarding children's oral health in Kuwait. *Subjects and Methods.* A nationwide cross-sectional survey was distributed to pediatricians and pediatric residents practicing in Kuwait. Data concerning demographic variables, knowledge on dental caries preventive measures, current anticipatory guidance, and experience with dental problems and oral care were collected using online and paper-based surveys.

**Results:**

A total of 230 completed the survey with a response rate of 50.1%. Most respondents (81%) had noticed dental caries in children more frequently. Two-thirds of respondents felt confident in detecting dental caries. However, more than two-thirds were unfamiliar with preventive dental practices and the management of dental trauma. Only 16.5% of the respondents got satisfactory knowledge score on the preventive measures questions, and nearly 51% got satisfactory knowledge on the recent anticipatory guidance questions. No significant correlations were found between gender or years of practice and knowledge scores. Respondents' confidence in detecting caries was significantly associated with the knowledge score of the anticipatory guidance (*p*=0.003).

**Conclusion:**

Dental caries is an oral problem commonly encountered by many pediatricians and pediatric residents in Kuwait. The majority has adequate knowledge of the current anticipatory guidance of oral health issues, but they have insufficient knowledge levels on oral preventative measures. Adequate education and training in oral health are highly recommended.

## 1. Introduction

Early childhood caries (ECC) is a major worldwide infectious oral health problem that affects preschool children [1]. ECC occurs as a result of the interaction of many risk factors such as pathogenic microorganisms, sugary diets, poor oral hygiene, low socioeconomic status, and irregular dental visits [2, 3]. Despite recommendations for young children to visit a dentist by 12 months [2], many parents often take their children to the dentist only when a dental problem becomes severe and causes pain or discomfort [4].

Regarding global prevalence, the World Health Organization (WHO) reported that about 60–90% of preschool children are affected by ECC in most countries [5]. The prevalence remains high in developing countries (up to 70%) and among children of low-income and minority status in developed countries [6]. In Kuwait, the prevalence of ECC in 4- and 5-year-old children was 68% and 76%, respectively [7]. It was similar to the prevalence reported in some Middle Eastern countries [8, 9].

Oral health has a significant impact on children's general health [10, 11]. ECC often results in pain and swelling that lead to problems in eating, speaking, and aesthetics. Moreover, the consequences of ECC have been associated with high emergency room visits [12], high treatment costs [13], loss of school days [14], lessened learning ability [15], and reduced oral health-related quality of life [16].

Parents usually contact pediatricians several times during the child's early life [17]. Pediatricians are often the primary care providers of young children when ECC may develop. Therefore, pediatricians can play a vital role in improving the oral health of their child patients. They may provide preventive information, early caries risk assessment, early detection, and appropriate referrals that help in the child's growth and development [17]. Unfortunately, pediatricians receive insufficient education on oral health during their medical training [17, 18]. Therefore, they should seek to know the updated knowledge and information related to oral health preventive measures [19].

The increasing prevalence of preschool children's dental caries experience in Kuwait [7] would necessitate further attention to assess the overall pediatricians' knowledge, attitudes, and practices regarding children's oral health. Therefore, the current study aimed to evaluate the knowledge level and practice of pediatricians and pediatric residents regarding children's oral health in Kuwait.

## 2. Materials and Methods

This was a nationwide cross-sectional survey study approved by the ethical committee of Kuwait University Health Sciences Centre and the Ministry of Health in Kuwait. The study population was pediatricians and pediatric residents who provide health care for children in Kuwait. They were identified using the membership databases of the Kuwait Medical Association (KMA) and the Kuwait Pediatric Association (KPA). To get a medical license and practice in Kuwait, it is a must to be a member of KMA. There were a total of 532 registered members (residents, pediatricians) in the database. Based on the current data, the minimum required respondents were predicted to be 224, to have a confidence level of 95% with an estimated 5% margin of error.

A structured questionnaire was adapted from previous surveys in the literature [20–24]. Then, it was piloted with a group of ten pediatric residents and ten pediatricians who were not included in the study results. No further changes were made. All items in the questionnaire were assessed for content and face validity among the pilot group on a Likert Scale. All participants found the questionnaire to be understandable and comprehensible. Brief information about children's dental caries experience in Kuwait and the study aims were provided on the cover page. The questionnaire included five sections. The first section obtained the sociodemographic characteristics of participants (e.g., age, gender, years of practice, practice sector, place of specialty degree, number of children they treat per day, and whether they receive any dental health-related subjects during medical school or residency). The second section consisted of six questions (yes/no) related to the participant's knowledge of preventive measures in children's oral health. The third section contained twelve knowledge-based questions (yes/no) on current anticipatory guidance and assessment practices during the childcare visit and was regarded as practising following the recommendations of the American Academy of Pediatrics (AAP) and AAPD. The fourth and fifth sections of the questionnaire addressed the participants' experience in pediatric dental problems and aspects of continuing education, respectively. The questionnaire was not expected to take longer than 10 minutes to complete.

The data were collected between January and March 2019. Recruitment was performed by distributing the questionnaire in two different formats. Online surveys, using Google Forms (https://www.google.com/forms), were sent to all registered KMA and KPA members' emails and WhatsApp (https://www.whatsapp.com). Two follow-up reminders to complete the survey were sent two weeks apart. The other format was paper surveys given to 314 registered attendees at the 4th International Pediatric Conference, which occurred in Kuwait in 2019. The conference participants were asked not to complete the survey if they had already returned the online surveys. Participation was anonymous and voluntary.

The knowledge level of participants on oral preventive measures and current anticipatory guidance and assessment practices was determined based on their total knowledge scores. The knowledge questions were scored as correct or incorrect according to the recommendations of the AAP and AAPD [2, 25–28]. A score of “1” was given for the correct answer and “0” for incorrect or unknown answers. A total knowledge score was calculated and then classified into three tertials [29]:

- Poor score: <50% of the correct answers
- Fair score: 50%–75% of the correct answers
- Satisfactory score: >75% of the correct answers

The data were entered into an Excel spreadsheet and then analyzed using Statistical Package for the Social Science version 20.0 software (SPSS Inc., Chicago, Ill., USA). Descriptive statistics (frequency, percentage, and mean) were determined. The chi-square test was used to determine the associations between the categorical variables. All *p* values <0.05 were considered statistically significant.

## 3. Results

**Figure 1:**
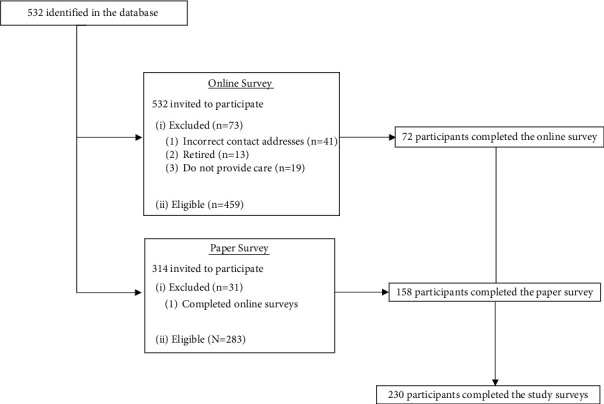
Flow chart of survey study participants.

Of the 532 registered members in the database, all invited to take part in the online study survey, 41 were returned because of incorrect email addresses and phone numbers (Figure 1). Of the remaining 491 members with valid addresses, 13 reported that they had retired and 19 reported that they did not provide care for young children (surgical specialties or administrators). Therefore, out of 459 eligible members, a total of 72 online surveys were completed. For the paper survey, out of 314 conference attendees, 31 reported their completion of the online survey. Of the 283 eligible attendees, 158 agreed to participate and completed the paper survey (response rate 55.8%). Therefore, a total of 230 agreed and completed online and paper surveys with a response rate of 50.1%.

Table 1 shows the sociodemographic characteristics of the study participants. The study sample included 194 pediatricians (84.3%) and 36 pediatric residents (15.7%). There were 127 female respondents (55.2%) and 103 male respondents (44.8%). More than a third of the respondents (37.8%) were in (35 to 44 years) age, and only 12.2% were above 55 years old. Almost one-third of the respondents (28.7%) had more than twenty years of practice, and 12.6% of the respondents had less than five years of practice. Two-thirds of respondents (62.2%) work in the public sector. About half of the respondents (47%) reported that they see 10 to 25 patients daily. The majority of respondents (64.3%) were board-certified. When asked about studying any dental health-related subjects during the medical school or residency program, 66.5% and 72.2% of respondents denied, respectively.

**Figure 2:**
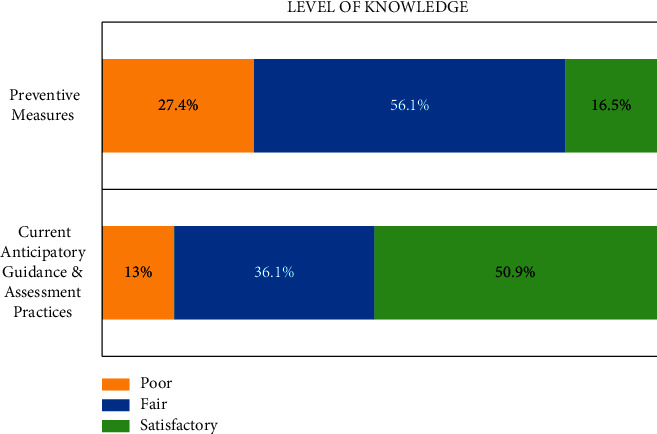
Participants' level of knowledge (%) on preventive measures and current anticipatory guidance and assessment practices.

The knowledge level of participants on the oral preventive measures was investigated and described in Table 2. Most respondents were aware that the baby bottle is not the only cause of ECC (62.2%), and the recommended age for the child's first dental visit is 1 (61.7%). Around half of the respondents knew that a 3-month-old child did not require fluoride supplementation (47%), bacterial transmission can occur from mother to child (55.2%), dental sealants are not often applied to primary teeth (50.9%), and fluoridated toothpaste should be used to children less than 3 years old (47.9%). Only 16.5% of respondents (*n* = 38) correctly answered all six questions and got a satisfactory level of knowledge. While more than half of the respondents (56.1%) got fair knowledge, followed by a third of the respondents (27.4%) with a poor knowledge score (Figure 2). There was no significant association between knowledge level and gender (*χ*^2^ (2) = 0.303; *p*=0.859) or years of practice (*χ*^2^ (2) = 10.5, *p*=0.230).

Regarding the participants' knowledge on the current anticipatory guidance and assessment practices (Table 3), the majority claimed that they inquired information about the infant's bottle use at bedtime (79.6%) and children's oral habits (65.2%), and they examined children's teeth for dental caries (72.2%). Furthermore, counseled on dental visits (67.4%), the importance of tooth brushing (73.9%), and children's sugar consumption (74.3%). Only half of the respondents considered prescribing sugar-free syrup (47.4%), and about 39% counseled the use of mouthguards during sports for schoolchildren. In addition, when asked about their opinion on specific dental practices to be part of the routine well-childcare visit, most respondents (85%) agreed to assess the dental problems and counsel on their prevention (Table 3). Besides, two-thirds (60.9%) acknowledged referring the child to a dentist at age 1. Additionally, half of them (54.8%) considered applying fluoride varnish in the office. Overall, 50% of participants got satisfactory knowledge levels of the current anticipatory guidance and assessment practices (shown in Figure 1). Neither gender (*χ*^2^ (2) = 0.767, *p*=0.681) nor years of practice (*χ*^2^ (2) = 8.7, *p*=0.368) had significant associations with the knowledge level.

The participants' experiences with dental problems and dental care are presented in Table 4. Nearly eighty percent of the respondents had noticed early childhood caries in young children on a monthly (43.5%) and a weekly basis (37.8%) in their practice. Moreover, dental caries in school-aged children were noticed more frequently by half of the respondents (51.3%) on a weekly basis. Many respondents (63.5%) were confident in detecting dental caries. Of those, 70% got a satisfactory knowledge level on the current anticipatory guidance and assessment practices compared to the unconfident respondents (30%, *χ*^2^ (2) = 11.5, *p*=0.003). In contrast, only 19.2% of those confident practitioners got a satisfactory knowledge level of preventive measures. There was no significant effect of practice years on the confidence level (*χ*^2^ (4) = 5.1, *p*=0.271). Many participants were unfamiliar with preventive dental practices such as fluoride varnish (68.7%) and dental sealants (73.9%) or with the management of dental trauma (74.8%).

Regarding the information source of oral health, 30.9% of respondents received oral health information. The internet was the primary information source (21.7%), followed by continuing education courses (12.2%) and books (11.3%, Table 5). Few respondents got information from scientific journals (5.7%). The necessity of having a continuing education course regarding children's oral health has been strongly agreed by respondents (85.2%, Table 5).

## 4. Discussion

Many surveys have been conducted internationally to investigate the role of non-dental practitioners in improving children's oral health outcomes [28]. Although the prevalence of dental caries in children is extremely high in Kuwait, to our knowledge, this is the first nationwide survey to investigate the pediatricians' knowledge and awareness regarding children's oral health.

Oral health education differed remarkably among several countries, and it was mainly delivered through continuing education and practical experience [30]. The current study showed that most respondents did not receive oral health education in medical school (66.5%) or residency programs (72.2%). A similar finding was reported (67%) in a recent European study [30], and even a higher percentage (90.4%) was documented in neighboring Saudi Arabia [31]. Inadequate knowledge or training on oral health issues may be a barrier for pediatricians to promote children's oral health effectively [21].

In the current survey, deficits of key knowledge related to dental caries preventive measures were spotted among half of the pediatricians. They were unaware of the transmission of cariogenic bacteria from mother to child and the international recommendations on specific interventions such as fluoride therapy and dental sealants. Other studies have documented similar findings in the knowledge of preventive measures [31–35], but a better knowledge of transmission was reported in the US and European studies [20, 30]. Regarding the first dental visit, AAP and AAPD recommend that all children have their initial dental visit no later than their first birthday [2]. Two-thirds of respondents in the current survey supported the dental referral of children by one year of age. They believed that it should be performed during the child's physical examination. This result was high compared to what was previously reported [20, 30–35]. According to other researchers, half of their responders recommended children's first dental visits be between the ages of 1 and 3 [21, 22, 29, 30]. Despite many pediatricians being aware of the recommended referral, Karasz et al. found that few managed to complete the referral [36].

Most pediatricians in the present study claimed that they assess children's teeth and include anticipatory guidance on oral health issues in their well-childcare visits. Variable rates were previously reported for the examination of children's teeth. Rates ranged from 90% in the US and Canadian studies [21, 26] to 60% in the Saudi and Emirati studies [31, 35] and lower rates in some Indian studies [32, 33]. Most pediatricians in the present survey (85%) believed that assessing dental problems and counseling on their prevention should be part of the routine childcare visit. Two-thirds (64%) of our respondents felt confident enough to detect dental caries. Studies conducted in the US, Europe, and India found similar results [21, 29, 30, 32]. Of note in the present study, no significant effect of years of practice was shown on the pediatricians' confidence in detecting caries. However, Hadjipanayis et al. found that experienced pediatricians (>10 years in practice) felt confident about identifying dental caries [30].

Around half of the surveyed pediatricians agreed that they should apply fluoride varnish during the routine childcare visit. Lewis et al. [21] found that although 21% of pediatricians agreed that the application of fluoride varnish should be a part of well-childcare, only 4% of them provided that. Pediatricians can play a vital role in preventing dental caries by offering the application of fluoride varnish in their practice [37]. It is an effortless procedure and doesn't require special training or operatory.

Relatively half of the pediatricians in the present study were likely to consider sugar-free syrup prescriptions. The result was inconsistent with Girish Babu et al.'s study [38]. The finding was not investigated in previous similar studies [21, 26, 29–35]. In general, many pediatricians believe that sugar-free medications are not as sweet as sugar-containing medications and are more expensive [38, 39].

With the increased participation by children in different sports, the risk of orofacial injuries is rising as a shared concern. About thirty percent of oral injuries in children have been reported to occur during sports activities [40]. AAPD and Academy for Sports Dentistry recommend using mouthguards for all children engaged in competitive sports [41]. In the current study, more than two-thirds were not aware of the management of dental trauma. Also, a few respondents (38%) counseled using sports mouthguards for schoolchildren. Educating pediatricians on the need for and use of mouthguards for children who engage in sports is essential.

Overall, half of the respondents in the current study demonstrated fair knowledge of preventive measures and satisfactory knowledge of anticipatory guidance and assessment practices. This might be explained by the fact that many respondents were board-certified and followed the AAP guidelines. However, the findings still imply significant gaps in preventive oral health and dental trauma topics. In our study, years of practice have not shown significant associations with investigated knowledge levels. Most of our respondents had eleven or more years of experience. Interestingly, Oge et al. found that years of experience were linked to a major decline in oral health knowledge [42].

In the present study, both paper-based surveys and online-based surveys were used. The conducted online surveys produced lower response rates compared to the paper-based surveys. The mixed-mode survey administration has been found to produce the highest response rate but at a considerably greater cost [43]. The respondents in the current study appeared to have a particular interest in the survey results and might have felt a professional obligation to complete the survey. The majority showed their need for further continuing education courses on the child's oral health.

There are some limitations in the current study. As with any survey, there is the potential for response bias. Although the response rate of 50.1% is consistent with previous surveys [20, 29], the nonrespondents possibly had other experiences and opinions regarding oral health in pediatric practice. Also, this study did not include family physicians since most of them work in polyclinics and often refer children's cases to pediatricians to evaluate-the rest work in administrative jobs. Even though the current survey research has validity problems in which the provided answers may not reflect the accurate participants' knowledge and practice, the findings of this survey would establish baseline data about pediatricians' oral health knowledge in Kuwait. In addition, it would be the foundation for the Kuwaiti Board of Pediatrics and local authorities to uphold good oral healthcare by running educational courses on different oral health aspects. Also, the current data generated from Kuwait, together with data from Saudi Arabia [31] and UAE [35], would give a clearer picture to the authorities of the Gulf Cooperation Council (GCC) in healthcare. The collaboration between the medical and dental sectors is a must to promote children's oral health. Further research is required to explore pediatricians' attitudes and practice on oral health prevention and orofacial trauma topic after implementing educational courses.

## 5. Conclusion

The current study shows that early childhood caries is an oral problem commonly encountered by many pediatricians and pediatric residents in Kuwait. Most of them have insufficient knowledge levels on the preventative measures of dental caries and dental trauma. However, they have adequate knowledge levels on the current anticipatory guidance of children's oral health. Many pediatricians were willing to perform appropriate oral assessments and dental referrals as well as fluoride varnish applications. Continuing oral health education courses during pediatric training programs is required to ensure high-quality care for children locally and internationally.

## Figures and Tables

**Table 1:** Socio-demographic characteristics of the study participants.

Characteristic	Total *N* = 230	Percentage (%)
*Gender*		
Female	127	55.2
Male	103	44.8
*Age Group*		
25-34	56	24.3
35-44	87	37.8
45-54	59	25.7
>55	28	12.2
*Current Position*		
Pediatricians	194	84.3
Pediatric Residents	36	15.7
*Years of Practice*		
<5	29	12.6
6-10	38	16.5
11-15	60	26.1
16-20	37	16.1
>20	66	28.7
*Practice Sector*		
Public Sector	143	62.1
Private Sector	39	17
Academic	8	3.5
Academic and public	22	9.6
Academic and private	18	7.8
Number of patients per day		
<10	67	29.1
10-25	108	47
>25	55	23.9
*Board-certification*		
Yes	148	64.3
No	82	35.7
*Receiving dental subjects in medical school*		
Yes	77	33.5
No	153	66.5
*Receiving dental subjects in residency program*		
Yes	64	27.8
No	166	72.2

**Table 2:** Responses to knowledge questions on preventive measures.

Questions	Correct response	Agreed *N* (%)	Disagreed *N* (%)	Don't know *N* (%)
Only bottle-fed children get early childhood caries (baby bottle tooth decay)	False	82 (35.6)	143 (62.2)	5 (2.2)
A 3-month-old baby living in non-fluoridated area needs fluoride supplementation	False	118 (51.3)	108 (47.0)	4 (1.7)
Dental decay-causing bacteria can be transmitted between mother and child	True	127 (55.2)	98 (42.6)	5(2.2)
Dental sealants are usually applied to the child's primary teeth	False	109 (47.4)	117 (50.9)	4 (1.7)
The recommended age for the child's first dental visit is at 1-year-old	True	142 (61.8)	84 (36.5)	4 (1.7)
Fluoridated toothpaste should not be used in children less than 3 years of age	False	116 (50.4)	110 (47.9)	4 (1.7)

**Table 3:** Current anticipatory guidance and assessment practices on the role of pediatricians in promoting oral health.

At a well-childcare visit in your practice, do you likely perform the following?	Likely *N* (%)
Inquire about infant's feeding practices and bottle use at bedtime	183 (79.6)
Examine child's teeth for dental cavities	166 (72.2)
Counsel on going to dentists	155 (67.4)
Counsel on the importance of toothbrushing	170 (73.9)
Counsel on child's sugar consumption	171 (97.3)
Consider sugar free syrup prescriptions	109 (47.4)
Counsel on the use of mouth guard during sports for school children	89 (38.7)
Inquire about child's oral habits	150 (65.2)

Should the following be a part of the routine well-childcare visit?	Agreed *N* (%)
Assessment for dental problems during physical examination	196 (85.2)
Counseling on the prevention of dental problems	195 (84.8)
Application of fluoride varnish	126 (54.8)
Referral to the dentist at 12 months of age	140 (60.9)

**Table 4:** Experience of the participants with dental problems and dental care.

Questions	*N* (%)
*How often do you notice* “*Early childhood caries*” *in young children*?	
At least once a month	100 (43.5)
At least once a week	87 (37.8)
I don't check children's teeth	43 (18.7)
*How often do you notice* “*Dental Caries*” *in school-aged children*?	
At least once a month	73 (31.7)
At least once a week	118 (51.3)
I don't check children's teeth	39 (17.0)
Are you confident in detecting dental caries?^*∗*^	146 (63.5)
Are you familiar enough with “fluoride varnish” for preventive dental care?^*∗*^	72 (31.3)
Are you familiar enough with “dental sealants” for preventive dental care?^*∗*^	60 (26.1)
Are you familiar enough with the management of dental trauma?^*∗*^	58 (25.2)

^*∗*^Yes answers only

**Table 5:** Aspects of continuing education among study participants.

Questions	*N* (%)
Are you receiving any information on oral health?	
*Yes*	71 (30.9)
Scientific journals	13 (5.7)
Continuing education	28 (12.2)
Brochures or pamphlets	12 (5.2)
Internet	50 (21.7)
Books	26 (11.3)
Others	9 (3.9)

Need for continuing education course regarding child's oral health?	
*Yes*	196 (85.2)
Diagnosis only	51 (22.2)
Management only	38 (16.5)
Prevention only	68 (29.6)
All	144 (62.6)

## Data Availability

Raw data have been stored securely at the Kuwait University Dental School. All the data generated or analyzed during the current study are included in this published article. The data are available on request to the corresponding author.

## References

[1] Anil S., Anand P. S. (2017). Early childhood caries: prevalence, risk factors, and prevention. *Frontiers in Pediatrics*.

[2] American Academy of Pediatric Dentistry (2020). Periodicity of examination, preventive dental services, anticipatory guidance/counseling, and oral treatment for infants, children, and adolescents. *The Reference Manual of Pediatric Dentistry*.

[3] Seow W. K. (2018). Early childhood caries. *Pediatric Clinics of North America*.

[4] Agostini F. G., Flaitz C. M., Hicks M. J. (2001). Dental emergencies in a university-based pediatric dentistry postgraduate outpatient clinic: a retrospective study. *American Society of Dentistry for Children*.

[5] Petersen P. E., Bourgeois D., Ogawa H., Estupinan-Day S., Ndiaye C. (2005). The global burden of oral diseases and risks to oral health. *Bulletin of the World Health Organization*.

[6] Congiu G., Campus G., Lugliè P. F. (2014). Early childhood caries (ECC) prevalence and background factors: a review. *Oral Health & Preventive Dentistry*.

[7] Al-Mutawa S. A., Shyama M., Al-Duwairi Y., Soparkar P. (2006). Dental caries experience of Kuwaiti school children. *Community Dental Health*.

[8] Azizi Z. (2014). The prevalence of dental caries in primary dentition in 4- to 5-year-old preschool children in northern Palestine. *International Journal of Dentistry*.

[9] El-Nadeef M. A., Hassab H., Al-Hosani E. (2010). National survey of the oral health of 5-year-old children in the United Arab Emirates. *Eastern Mediterranean Health Journal*.

[10] Krol D. M. (2003). Dental caries, oral health, and pediatricians. *Current Problems in Pediatric and Adolescent Health Care*.

[11] Sheiham A. (2006). Dental caries affects body weight, growth and quality of life in preschool children. *British Dental Journal*.

[12] Ladrillo T. E., Hobdell M. H., Caviness A. C. (2006). Increasing prevalence of emergency department visits for pediatric dental care, 1997–2001. *Journal of the American Dental Association*.

[13] Griffin S. O., Gooch B. F., Beltrán E., Sutherland J. N., Barsley R. (2000). Dental services, costs, and factors associated with hospitalization for medicaid-eligible children, Louisiana 1996-97. *Journal of Public Health Dentistry*.

[14] Edelstein B. L., Reisine S. (2015). Fifty-one million: a mythical number that matters. *Journal of The American Dental Association*.

[15] Blumenshine S. L., Vann W. F., Gizlice Z., Lee J. Y. (2008). Children’s school performance: impact of general and oral health. *Journal of Public Health Dentistry*.

[16] Singh N., Dubey N., Rathore M., Pandey P. (2020). Impact of early childhood caries on quality of life: child and parent perspectives. *Journal of Oral Biology and Craniofacial Research*.

[17] Krol D. M. (2010). Children’s oral health and the role of the pediatrician. *Current Opinion in Pediatrics*.

[18] Gupta S. K., Gupta S., Gojanur S., Kour G., Singh K., Rani P. (2019). Pediatricians’ view on early childhood caries and oral health in a north region of India: a cross-sectional study. *Journal of Family Medicine and Primary Care*.

[19] Weatherspoon D. J., Horowitz A. M., Kleinman D. V. (2016). Maryland physicians’ knowledge, opinions, and practices related to dental caries etiology and prevention in children. *Pediatric Dentistry*.

[20] Lewis C. W., Grossman D. C., Domoto P. K., Deyo R. A. (2000). The role of the pediatrician in the oral health of children: a national survey. *Pediatrics*.

[21] Lewis C. W., Boulter S., Keels M. A. (2009). Oral health and pediatricians: results of a national survey. *Academic Pediatrics*.

[22] Sezer R. G., Paketci C., Bozaykut A. (2013). Paediatricians’ awareness of children’s oral health: knowledge, training, attitudes and practices among Turkish paediatricians. *Paediatrics and Child Health*.

[23] Prakash P., Lawrence H. P., Harvey B. J., McIsaac W. J., Limeback H., Leake J. L. (2006). Early childhood caries and infant oral health: paediatricians’ and family physicians’ knowledge, practices and training. *Paediatrics and Child Health*.

[24] Herndon J. B., Tomar S. L., Lossius M. N., Catalanotto F. A. (2010). Preventive oral health care in early childhood: knowledge, confidence, and practices of pediatricians and family physicians in Florida. *The Journal of Pediatrics*.

[25] American Academy of Pediatric Dentistry (2020). Policy on oral health care programs for infants, children, adolescents, and individuals with special health care needs. *The Reference Manual of Pediatric Dentistry*.

[26] Steve H., Braun P., James D. I., Kristen N., Robert J. S., American Academy of Pediatrics Committee on Native American Child Health and Section on Oral Health Canadian Paediatric Society First Nations Inuit and Metis Health Committee (2021). Early childhood caries in indigenous communities. *Pediatrics*.

[27] American Academy of Pediatric Dentistry (2020). Fluoride therapy. *The Reference Manual of Pediatric Dentistry*.

[28] Dickson-Swift V., Kenny A., Gussy M., McCarthy C., Bracksley-O’Grady S. (2020). The knowledge and practice of pediatricians in children’s oral health: a scoping review. *BMC Oral Health*.

[29] Kumar P., Kumar P., Dixit A., Gupta V., Singh H., Sargaiyan V. (2014). Cross-sectional evaluation of awareness of prevention of dental caries among general pediatricians in Ghaziabad district, India. *Annals of Medical and Health Sciences Research*.

[30] Hadjipanayis A., Grossman Z., Del Torso S., Michailidou K., Van Esso D., Cauwels R. (2018). Oral health training, knowledge, attitudes and practices of primary care paediatricians: a European survey. *European Journal of Pediatrics*.

[31] Alshunaiber R., Alzaid H., Meaigel S., Aldeeri A., Adlan A. (2019). Early childhood caries and infant’s oral health; pediatricians’ and family physicians’ practice, knowledge and attitude in Riyadh city, Saudi Arabia. *The Saudi Dental Journal*.

[32] Murthy G. A., Mohandas U. (2010). The knowledge, attitude and practice in prevention of dental caries amongst pediatricians in Bangalore: a cross-sectional study. *Journal of Indian Society of Pedodontics and Preventive Dentistry*.

[33] Poornima P., Bajaj M., Nagaveni N. B., Roopa K. B., Neena I. E., Bharath K. P. (2015). Evaluation of the knowledge, attitude and awareness in prevention of dental caries amongst paediatricians. *International Journal of Community Medicine and Public Health*.

[34] Golubović L., Selimović-Dragaš M., Kobašlija S., Huseinbegović A. (2020). The role of the pediatricians in dental caries prevention in Montenegro: the knowledge, attitude and practice. *Balkan Journal of Dental Medicine*.

[35] Aburahima N., Hussein I., Kowash M., Alsalami A., Al Halabi M. (2020). Assessment of paediatricians’ oral health knowledge, behaviour, and attitude in the United Arab Emirates. *International Journal of Dentistry*.

[36] Karasz A., Patel V., Ranasinghe S., Chaudhuri K., McKee D. (2014). Preventing caries in young children of immigrant Bangladeshi families in New York: perspectives of mothers and paediatricians. *Community Dental Health*.

[37] Geissler K. H., Dick A. W., Goff S. L., Whaley C., Kranz A. M. (2021). Dental fluoride varnish application during medical visits among children who are privately insured. *JAMA Network Open*.

[38] Girish Babu K. L., Doddamani G. M., Kumaraswamy N. L. R. (2017). Knowledge, attitude, and practice of pediatricians regarding pediatric liquid medicaments. *European Journal of Dermatology*.

[39] Bawazir O. A., Alsuwayt B., Alqahtani W., Al-Dhafiri A., Al-Shamrani M. (2014). Knowledge, attitude and practice of pediatricians and pharmacists in Riyadh City toward the use of sugar free medications. *The Journal of Contemporary Dental Practice*.

[40] Gassner R., Tuli T., Hachl O., Moreira R., Ulmer H. (2004). Craniomaxillofacial trauma in children: a review of 3,385 cases with 6,060 injuries in 10 years. *Journal of Oral and Maxillofacial Surgery*.

[41] American Academy of Pediatric Dentistry (2021). Policy on prevention of sports-related orofacial injuries. *The Reference Manual of Pediatric Dentistry*.

[42] Oge O. A., Douglas G. V. A., Seymour D., Adams C., Csikar J. (2018). Knowledge, attitude and practice among health visitors in the United Kingdom toward children’s oral health. *Public Health Nursing*.

[43] Greenlaw C., Brown-Welty S. (2009). A comparison of web-based and paper-based survey methods: testing assumptions of survey mode and response cost. *Evaluation Review*.

